# PRINS: scalable model inference for component-based system logs

**DOI:** 10.1007/s10664-021-10111-4

**Published:** 2022-04-12

**Authors:** Donghwan Shin, Domenico Bianculli, Lionel Briand

**Affiliations:** 1 University of Luxembourg, Esch-sur-Alzette, Luxembourg; 2 University of Ottawa, Ottawa, ON Canada

**Keywords:** Logs, Model inference, Component-based system

## Abstract

Behavioral software models play a key role in many software engineering tasks; unfortunately, these models either are not available during software development or, if available, quickly become outdated as implementations evolve. Model inference techniques have been proposed as a viable solution to extract finite state models from execution logs. However, existing techniques do not scale well when processing very large logs that can be commonly found in practice. In this paper, we address the scalability problem of inferring the model of a component-based system from large system logs, without requiring any extra information. Our model inference technique, called *PRINS*, follows a divide-and-conquer approach. The idea is to first infer a model of each system component from the corresponding logs; then, the individual component models are merged together taking into account the flow of events across components, as reflected in the logs. We evaluated *PRINS* in terms of scalability and accuracy, using nine datasets composed of logs extracted from publicly available benchmarks and a personal computer running desktop business applications. The results show that *PRINS* can process large logs much faster than a publicly available and well-known state-of-the-art tool, without significantly compromising the accuracy of inferred models.

## Introduction

Behavior models of software system components play a key role in many software engineering tasks, such as program comprehension (Cook and Wolf 1998), test case generation (Fraser and Walkinshaw 2012), and model checking (Clarke et al. 2018). Unfortunately, such models are either scarce during software development or, if available, quickly become outdated as the corresponding implementations evolve, because of the time and cost involved in generating and maintaining them (Walkinshaw et al. 2010).

One possible way to overcome the lack of software models is to use *model inference* techniques, which extract models—typically in the form of Finite State Machine (FSM)—from execution logs. Although the problem of inferring a minimal FSM is NP-complete (Gold 1967), there have been several proposals of polynomial-time approximation algorithms to infer FSMs (Biermann and Feldman 1972; Beschastnikh et al. 2011) or richer variants, such as gFSM (guarded FSM) (Walkinshaw et al. 2016; Mariani et al. 2017) and gFSM extended with transition probabilities (Emam and Miller 2018), to obtain relatively faithful models.

Though the aforementioned model inference techniques are fast and accurate enough for relatively small programs, all of them suffer from scalability issues, due to the intrinsic computational complexity of the problem. This leads to out-of-memory errors or extremely long, impractical execution time when processing very large logs (Wang et al. 2015) that can be commonly found in practice. A recent proposal (Luo et al. 2017) addresses scalability using a distributed FSM inference approach based on MapReduce. However, this approach requires to encode the data to be exchanged between mappers and reducers in the form of key-value pairs. Such encoding is application-specific; hence, it cannot be used in contexts—like the one in which this work has been performed—in which the system is treated as a black-box (i.e., the source code is not available), with limited information about the data recorded in the system logs.

In this paper, we address the scalability problem of inferring a system model from the logs recorded during the execution (possibly multiple executions) of a system composed of multiple “components” (hereafter called *component-based system*), without requiring any extra information other than the logs. In this paper, we use the term “component” in a broad sense: the large majority of modern software systems are composed of different types of “components”, such as modules, classes, and services; in all cases, the resulting system decomposition provides a high degree of modularity and separation of concerns. Our goal is to efficiently infer a system model that captures not only the components’ behaviors but also the flow of events across the components as reflected in the logs.

Our approach, called *PRINS*, follows a *divide-and-conquer* strategy: we first infer a model of each component from the corresponding logs using a state-of-the-art model inference technique, and then we “stitch” (i.e., we do a peculiar type of merge) the individual component models into a system-level model by taking into account the interactions among the components, *as reflected in the logs*. The rationale behind this idea is that, though existing model inference techniques cannot deal with the size of all combined component logs, they can still be used to infer the models of individual components, since their logs tend to be sufficiently small. In other words, *PRINS* alleviates the scalability issues of existing model inference techniques by limiting their application to the smaller scope defined by component-level logs.

We implemented *PRINS* in a prototype tool, which internally uses MINT (Walkinshaw et al. 2016), the only publicly available state-of-the-art technique for inferring gFSMs, to infer the individual component models. We evaluate the scalability (in terms of execution time) and the accuracy (in terms of recall and specificity) of *PRINS* in comparison with MINT (to directly infer system models from system logs), on nine datasets composed of logs extracted from publicly available benchmarks (He et al. 2020) and a personal computer (PC) running desktop business applications on a daily basis. The results show that *PRINS* is significantly more scalable than MINT and can even enable model inference when MINT leads to out-of-memory failures. It also achieves higher specificity than MINT (with a difference ranging between -3.1 pp and + 34.9 pp, with pp=percentage points) while achieving lower recall than MINT (with a difference ranging between -23.5 pp and + 0 pp). Through a detailed analysis, we determined that a lower recall for *PRINS* only happens when logs are inadequate to infer accurate models, using any of the techniques. We also propose a simple and practical metric for engineers to easily predict (and thus improve) such cases before running model inference. With adequate logs, *PRINS* therefore provides a comparable or even better accuracy.

To summarize, the main contributions of this paper are:

- the *PRINS* approach for taming the scalability problem of inferring the model of a component-based system from the individual component-level logs, when no additional information is available;
- the novel *stitching* algorithm that “combine” individual component models together taking into account the flow of events across components as recorded in logs;
- a publicly available implementation of *PRINS* (see Section 5.8);
- the empirical evaluation, in terms of scalability and accuracy, of *PRINS* and its comparison with the state-of-the-art model inference tool.

The rest of the paper is organized as follows. Section 2 gives the basic definitions of logs and models that will be used throughout the paper. Section 3 illustrates the motivating example. Section 4 describes the different stages of *PRINS*. Section 5 reports on the evaluation of *PRINS*. Section 6 discusses related work. Section 7 concludes the paper and provides directions for future work.

## Background

This section provides the basic definitions for the main concepts that will be used throughout the paper.

### Logs

A *log* is a sequence of log entries; a *log entry* contains a timestamp (recording the time at which the logged event occurred), a component (representing the name of the component where the event occurred), and a log message (with run-time information related to the logged event). A log message is typically a block of free-form text that can be further decomposed (Zhu et al. 2019; He et al. 2017; Messaoudi et al. 2018; El-Masri et al. 2020) into an event template, characterizing the event type, and the parameter values of the event, which are determined at run time. For example, given the log entry “15:37:56 - Master - end (status=ok)”, we can see that the event end of the component Master occurred at timestamp 15:37:56 with the value ok for parameter status.

More formally, let *C* be the set of all components of a system, *ET* be the set of all events that can occur in the system, *V* be the set of all mappings from event parameters to their concrete values, for all events *et* ∈ *ET*, and *L* be the set of all logs retrieved for the system; a log *l* ∈ *L* is a sequence of log entries $\langle e_{1}, e_{2}, \dots , e_{n} \rangle $, with *e*_*i*_ = (*ts*_*x*_,*cm*_*i*_,*et*_*i*_,*v*_*i*_), ${ts}_{i}\in \mathbb {N}$, *cm*_*i*_ ∈ *C*, *et*_*i*_ ∈ *ET*, and *v*_*i*_ is a vector of parameter values over *V*. To denote individual log entries, we use the notation *e*_*i*,*j*_ for the *i*-th log entry in the *j*-th execution log.

### Models

In this paper, we represent the models inferred for a system for a component as guarded Finite State Machines (gFSMs). Informally, a gFSM is an “extended” finite state machine whose transitions are triggered by the occurrence of an event and are guarded by a function that evaluates the values of the event parameters.

More formally, let *ET* and *V* be defined as above. A gFSM is a tuple *m* = (*S*,*ET*,*G*,*δ*,*s*_0_,*F*), where *S* is a finite set of states, *G* is a finite set of guard functions of the form *g*: *V* →{*True*,*False*}, *δ* is the transition relation $\delta \subseteq S\times {ET} \times G \times S$, *s*_0_ ∈ *S* is the initial state, and $F\subseteq S$ is the set of final states. A gFSM *m* makes a guarded transition from a (source) state *s* ∈ *S* to a (target) state $s^{\prime }\in S$ when reading an input log entry *e* = (*ts*,*cm*,*et*,*v*), written as $s \xrightarrow {e} s^{\prime }$, if $(s,{et}, g, s^{\prime }) \in \delta $ and *g*(*v*) = *True*. We say that a guarded transition is *deterministic* if there is at most one target state for the same source state and the same log entry. Otherwise, it is *non-deterministic*. Based on this, we say that a gFSM is deterministic if all of its guarded transitions are deterministic; otherwise, the gFSM is non-deterministic. We say that a gFSM *m* *accepts* a log $l = \langle e_{1}, \dots , e_{n} \rangle $ if there exists a sequence of states $\langle \gamma _{0}, \dots , \gamma _{n} \rangle $ such that (1) *γ*_*i*_ ∈ *S* for $i=0,\dots ,n$, (2) *γ*_0_ = *s*_0_, (3) $\gamma _{i-1} \xrightarrow {e_{i}} \gamma _{i}$ for $i=1,\dots ,n$, and (4) *γ*_*n*_ ∈ *F*.

## Motivating Example

This section presents a simple example to motivate and demonstrate our work.

**Fig. 1:**
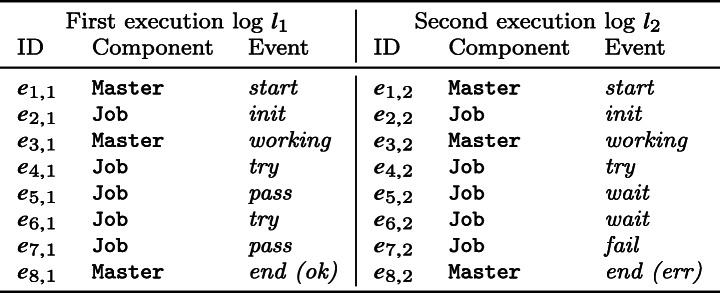
Running example logs *L*_S_ = {*l*_1_,*l*_2_}, inspired by Hadoop logs (He et al. 2020)

Let us consider an imaginary system composed of two components, Master and Job; Fig. 1 depicts the set of logs *L*_S_ = {*l*_1_,*l*_2_} recorded during the executions of the system. Entries in the logs are denoted using the notation introduced in Section 2.1; for instance, log entry *e*_8,1_ corresponds to the tuple (⋅,Master,*end*,[*ok*]), where the event is “*end*” and the value for its first (and only) parameter is “*ok*”. Notice that in Fig. 1 we use a short form (as in “*end (ok)*”) to indicate both an event and its parameter value; also, we omit timestamps in the running example logs as they are not used in our approach.

**Fig. 2:**
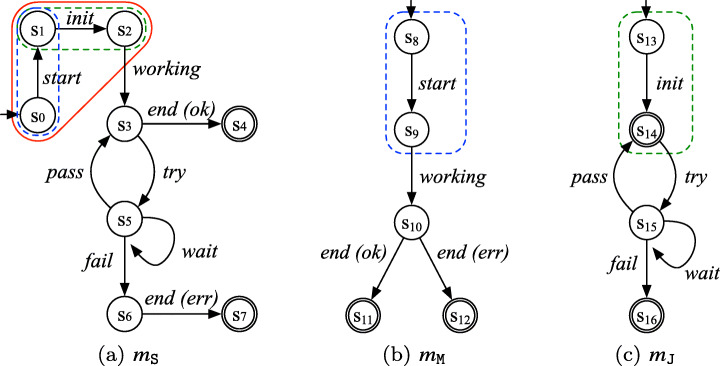
Models corresponding to the running example logs (*m*_S_: system model, *m*_M_: model for component Master, *m*_J_: model for component Job)

A software engineer is tasked with building a finite-state model of the system that accurately captures the behavior observed in the logs. However, the engineer cannot rely on the system source code since it is not available. This is the case, for example, where the system is mainly composed of heterogeneous, 3rd-party components for which neither the source code nor the documentation are available (Aghajani et al. 2019; Palmer and McAddis 2019; Rios et al. 2020). The only information about the system to which engineers have access is represented by the execution logs *L*_S_. To perform this task, the engineer uses a tool implementing one of the state-of-the-art *model inference techniques* (Walkinshaw et al. 2016; Mariani et al. 2017; Emam and Miller 2018) proposed in the literature; the tool takes as input the logs *L*_S_ and returns the system model *m*_S_ shown in Fig. 2a. Intuitively, we can see that *m*_S_ properly reflects the flow of events recorded in *L*_S_. However, when the engineer tries to execute the model inference tool on much larger logs of the same system, she observes that the tool does not terminate within a practical time limit (e.g., one day). Indeed, due to the intrinsic complexity of the model inference problem (Gold 1967), the time complexity of state-of-the-art model inference algorithms is polynomial (Lang et al. 1998; Emam and Miller 2018) in the size of the input logs.

To address this problem, the engineer decides to use our new approach, *PRINS*: it takes as input the logs *L*_S_ in Fig. 1 and returns the *same* system model *m*_S_ shown in Fig. 2a; the main difference with the tool used in the previous attempts is that *PRINS* takes considerably less time to yield a system model.

The main idea behind *PRINS* is to tackle the intrinsic complexity of model inference by means of a *divide-and-conquer* approach: *PRINS* uses existing model inference techniques to infer a model, not for the whole system but *for each component*. Figure 2b and c show the component models *m*_M_ and *m*_J_ for the Master and Job components, respectively. Component-level model inference is one of the main contributors to the significant reduction of the execution time achieved by *PRINS*. Furthermore, component-level model inference can be easily parallelized.

However, before yielding a system model, *PRINS* needs to properly “combine” the individual component models. In our running example, this means building the *m*_S_ model shown in Fig. 2 by “combining” the component models in *M*_*C*_ = {*m*_M_,*m*_J_} and shown in Fig. 2b and c. This is a challenging problem: we cannot simply concatenate or append the two component models together, because the result would not conform to the flow of events across the components recorded in the logs. In our running example logs, it is recorded that the event *start* of Master is immediately followed by the event *init* of Job. Such a flow of events recorded in the logs should be represented in the final system model produced by *PRINS*. To efficiently and effectively solve this problem, we propose a novel algorithm for *stitching* component models in the context of model inference.

## Scalable Model Inference

Our technique for scalable model inference follows a *divide-and-conquer* approach. The main idea is to first *infer* a model of each system component from the corresponding logs that are generated by the *projection* of system logs on the components; then, the individual component models are *stitched* together taking into account the flows of the events across the components, *as reflected in the logs*. We call this approach *PRINS* (*PR* ojection-*IN* ference-*S* titching). The rationale behind *PRINS* is that, though existing (log-based) model inference techniques cannot deal with the size of system logs, they can still be used to accurately infer the models of individual components, since their logs are sufficiently small for the existing model inference techniques to work. As anticipated in Section 3, the challenge is then how to “stitch” together the models of the individual components to build a system model that reflects not only the components’ behaviors but also the flow of events across the components, while preserving the accuracy of the component models. Tackling this challenge is our main contribution, as detailed in Section 4.3.

**Fig. 3:**
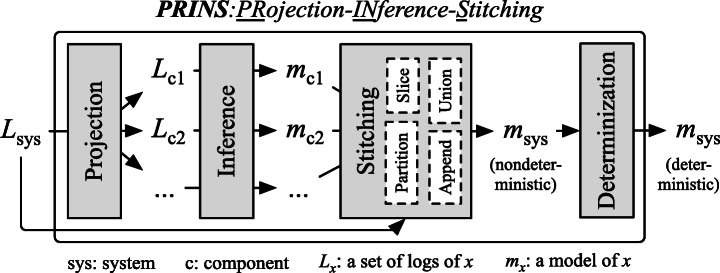
Overview of *PRINS*

Figure 3 outlines the workflow of *PRINS*. It takes as input the logs of the system under analysis, possibly coming from multiple executions; it returns a system model in the form of a gFSM. *PRINS* is composed of four main stages: *projection*, *inference*, *stitching*, and *determinization*. The projection stage produces a set of logs for each component from the input system logs. The component logs are then used to infer individual component models in the inference stage. The stitching stage combines the component models into a non-deterministic system model. Last, the determinization stage transforms the non-deterministic model into a deterministic model that is the output of *PRINS*. The four stages are described in detail in the following subsections.

We remark that *PRINS* does not require any extra information (e.g., source code and documentation) other than logs. Furthermore, we do not restrict logs to be produced by one thread/process: as one can see in the replication package for our evaluation (see Section 5.8), individual logs for many of our subject systems are already produced by multiple threads/processes (distinguished by tid/pid). Our only assumption is that log entries contain the name of the “component” that generated them. This assumption is realistic since this is common in practice, as shown in real logs (He et al. 2020). Also, as indicated in Section 1, we use the term “component” in a broad sense (e.g., modules, classes) to represent an architectural “part” of a system. Therefore, *PRINS* is applicable to any software system composed of multiple components as long as their behavior is recorded in logs.

### Projection

This stage generates a set of component logs—which will be used to infer a model for each component—from system logs. For instance, for our running example logs *L*_S_ = {*l*_1_,*l*_2_} shown in Fig. 1, we want to generate the set of logs for the Master component *L*_M_ = {〈*e*_1,1_,*e*_3,1_,*e*_8,1_〉, 〈*e*_1,2_,*e*_3,2_,*e*_8,2_〉}, and the set of logs for the Job component *L*_J_ = {〈*e*_2,1_,*e*_4,1_,*e*_5,1_,*e*_6,1_,*e*_7,1_〉,〈*e*_2,2_,*e*_4,2_,*e*_5,2_,*e*_6,2_,*e*_7,2_〉}. To achieve this, we define the *projection* operation as follows. Let *L* be a set of logs of a system and *C* be a set of components of the system; the projection of *L* for a component *c* ∈ *C*, denoted with *L*|*c*, is the set of logs obtained from *L* by removing all occurrences of log entries of all *c*^′^∈ *C* where *c*^′^≠*c*. For the running example, we have *L*_S_|Master = *L*_M_ and *L*_S_|Job = *L*_J_.

### Inference

This stage infers individual component models from the sets of component logs generated from the projection stage. This is straightforward because inferring a (component) model from a set of logs can be achieved using an off-the-shelf model inference technique. We remark that *PRINS* does not depend on any particular model inference technique, as long as it yields a deterministic FSM (or a deterministic gFSM^1^) as a resulting model. Also, *PRINS* can infer multiple component models in parallel because the inference processes of the individual component models are independent from each other. For the running example, using an off-the-shelf model inference tool like MINT (Walkinshaw 2018) on the logs in *L*_M_ and *L*_J_, we obtain models *m*_M_ (see Fig. 2b) and *m*_J_ (see Fig. 2c), respectively.

We want to note that the parallelization of component model inference is just a byproduct of using the divide-and-conquer approach enabled by the central component of *PRINS*: stitching (Section 4.3).

### Stitching

Individual component models generated from the inference stage are used in this *stitching* stage, which is at the core of *PRINS*. In this stage, we build a system model that captures not only the components’ behaviors inferred from the logs but also the flow of events across components as reflected in the logs. For the running example, this means building a model that is as “similar” as possible to *m*_S_, using models *m*_M_ and *m*_J_ as well as the input logs *L*_S_ reflecting the flow of events.

The idea of stitching comes from two important observations on the system and component models: (1) A system model is the composition of *partial models* of the individual components; this means that *partial behaviors* of components are *interleaved* in a system model. (2) The component partial models (included within a system model) are combined together (i.e., appended) *according to the flow of events recorded in logs*, since the system model must be able to accept the logs that were used to infer it.

For example, in the models shown in Fig. 2, we can see that the subgraph of *m*_S_, enclosed with a red solid line, contains two partial models: one, called $m_{\texttt {M}}^{p}$ and enclosed with a blue dashed line, extracted from *m*_M_ (including the states *s*_8_ and *s*_9_—mapped to *s*_0_ and *s*_1_ in *m*_S_—and the corresponding transition labeled with *start*) and the other, called $m_{\texttt {J}}^{p}$ and enclosed with a green dashed line, extracted from *m*_J_ (including the states *s*_13_ and *s*_14_—mapped to *s*_1_ and *s*_2_ in *m*_S_—and the corresponding transition labeled with *init*). Notice that the partial models $m_{\texttt {M}}^{p}$ and $m_{\texttt {J}}^{p}$ correspond to the partial (behaviors recorded in the) logs 〈*e*_1,1_〉 and 〈*e*_2,1_〉, respectively, that are determined by the interleaving of components in the system log *l*_1_ shown in Fig. 1. Furthermore, in *m*_S_, $m_{\texttt {J}}^{p}$ is appended to $m_{\texttt {M}}^{p}$, reflecting the fact that event *start* (from component Master) is immediately followed by *init* (from component Job) in the input logs.

Based on these observations, we propose a novel stitching technique that first “*slices*” individual component models into partial models according to the component interleavings shown in logs; then it “*appends*” partial models according to the flow of the events recorded in logs. However, the behaviors of components recorded in logs can be different from execution to execution (for instance, see the difference in terms of recorded events between *l*_1_ and *l*_2_ in our running example). To address this, we first build an intermediate, system-level model *for each execution* (i.e., for each log) and then merge these models together at the end.

The ***Stitch*** algorithm (whose pseudocode is shown in Algorithm 1) takes as input a set of logs *L*_*sys*_ and a set of component models *M*_*C*_; it returns a system model *m*_*sys*_ (built from the elements in *M*_*C*_) that accepts *L*_*sys*_. 
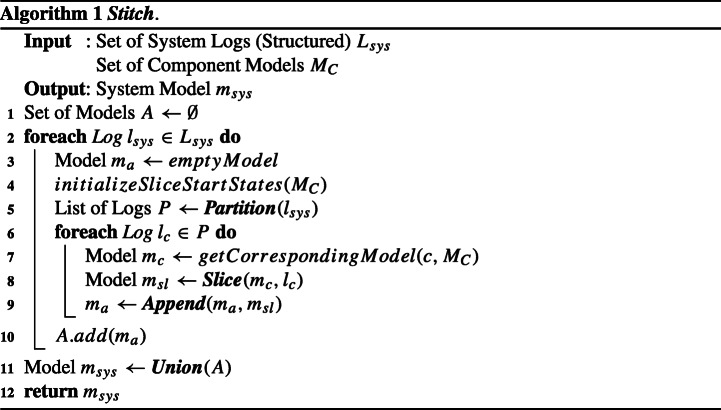


The algorithm builds a system-level model *m*_*a*_ for each system log *l*_*sys*_ ∈ *L*_*sys*_ (lines 2–10). To build *m*_*a*_ for a given *l*_*sys*_, the algorithm first initializes *m*_*a*_ as an empty model (lin3) and initializes the start states of all components models in *M*_*C*_ to their initial states (line 4). The algorithm then partitions *l*_*sys*_ into a list of logs *P*, each one corresponding to log entries of one component, according to the component interleavings shown in *l*_*sys*_ (using algorithm ***Partition*** at line 5, described in detail in Section 4.3.1). For each log *l*_*c*_ ∈ *P* (lines 6-9), the algorithm retrieves the component model *m*_*c*_ ∈ *M*_*C*_ of the component *c* that produced *l*_*c*_, slices it (using algorithm ***Slice*** at line 8, described in detail in Section 4.3.2) into a partial model *m*_*sl*_ that accepts *only* log *l*_*c*_, and then appends *m*_*sl*_ to *m*_*a*_ (using algorithm ***Append*** at line 9, described in detail in Section 4.3.3). During the iteration over the system logs in *L*_*sys*_, the resulting system-level models *m*_*a*_ are collected in the set *A*. Last, the models in *A* are combined into a single model *m*_*sys*_ (using algorithm ***Union*** at line 11, described in detail in Section 4.3.4). The algorithm ends by returning *m*_*sys*_ (line 12), inferred from all logs in *L*_*sys*_.

Before illustrating an example for ***Stitch***, let us first present the details of the auxiliary algorithms ***Partition***, ***Slice***, ***Append***, and ***Union***.

#### Partition

This algorithm takes as input a system log *l* (i.e., a sequence of log entries from various components); it partitions *l* into a sequence of logs *P*, where each log *l*_*c*_ ∈ *P* is the longest uninterrupted sequence of log entries produced by the same component, and returns *P*. By doing this, we can divide a system log into component-level logs, each of which represents the longest uninterrupted partial behavior for a component, while preserving the flow of events across components as recorded in the system log.

For instance, when the function takes as input the running example log *l*_1_, it returns $P = \langle l_{c,1}, l_{c,2}, \dots , l_{c,5} \rangle $ where *l*_*c*,1_ = 〈*e*_1,1_〉, *l*_*c*,2_ = 〈*e*_2,1_〉, *l*_*c*,3_ = 〈*e*_3,1_〉, *l*_*c*,4_ = 〈*e*_4,1_,*e*_5,1_,*e*_6,1_,*e*_7,1_〉, and *l*_*c*,5_ = 〈*e*_8,1_〉.

#### Slice

This algorithm (whose pseudocode is shown in Algorithm 2) takes as input a component model *m*_*c*_ and a component log *l*_*c*_; it returns a new model *m*_*sl*_, which is the sliced version of *m*_*c*_ and accepts only *l*_*c*_. 
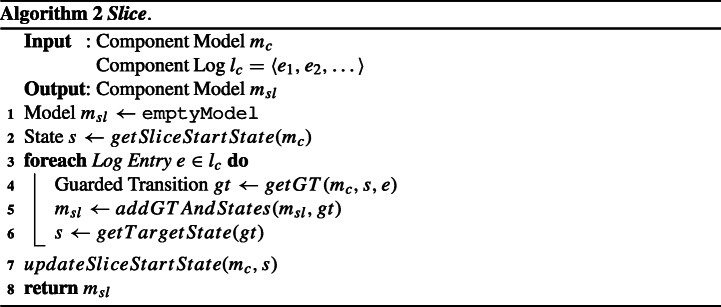


First, the algorithm retrieves the state of *m*_*c*_ that will become the initial state *s* of the sliced model *m*_*sl*_ (line 2). Upon the first invocation of ***Slice*** for a certain model *m*_*c*_, *s* will be the initial state of *m*_*c*_; for the subsequent invocations, *s* will be the last state visited in *m*_*c*_ when running the previous slice operations. Note that there is always only one last visited state because *m*_*c*_ is deterministic, as described in Section 4.2. Starting from *s*, the algorithm performs a run of *m*_*c*_ as if it were to accept *l*_*c*_ by iteratively reading each log entry *e* ∈ *l*_*c*_: the traversed states and guarded transitions of *m*_*c*_ are added into *m*_*sl*_ (lines 3-6). After the end of the iteration, the algorithm records the last state *s* visited in *m*_*c*_ (line 7), which is the (only one) final state of *m*_*sl*_ and will be used as the initial state of the next slice on *m*_*c*_. The algorithm ends by returning *m*_*sl*_.

**Fig. 4:**
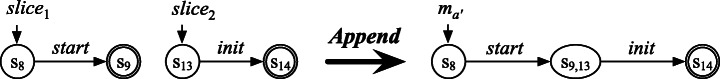
Illustration of appending two sliced models generated by ***Slice*** for *l*_*c*,1_ = 〈*e*_1,1_〉 and *l*_*c*,2_ = 〈*e*_2,1_〉. They are appended by ***Append***, resulting in $m_{a^{\prime }}$ that accepts 〈*e*_1,1_,*e*_2,1_〉

For example, let us consider the case where ***Slice*** is called with parameters *m*_*c*_ = *m*_M_ and *l*_*c*_ = 〈*e*_1,1_〉, and the slice start state returned by *getSliceStartState* for *m*_M_ is the initial state *s*_8_. Starting from *s* = *s*_8_, a run of *m*_M_ is performed: reading log entry *e*_1,1_ results in making the guarded transition to *s*_1_ in *m*_M_. This results in *m*_*sl*_ to include the guarded transition from *s*_8_ to *s*_9_ with label *start* as well as the states *s*_8_ and *s*_9_; the call to function *getTargetState* updates *s* to *s*_9_. Since there is no more log entry in *l*_*c*_, *s*_9_ is the final state of *m*_*sl*_ and is the slice start state for the next call to ***Slice*** for *m*_M_. The resulting *m*_*sl*_ is *slice*_1_ shown in Fig. 4.

#### Append

This algorithm takes as input two models *m*_*a*_ and *m*_*sl*_; it returns an updated version of *m*_*a*_ built by appending *m*_*sl*_ to the end of the original version of *m*_*a*_. If *m*_*a*_ is an empty model (i.e., when ***Slice*** is called for the first time after the initialization of *m*_*a*_ in line 3 in Algorithm 1), the algorithm simply returns *m*_*sl*_. Otherwise, the algorithm merges the final state of *m*_*a*_ and the initial state of *m*_*sl*_ and ends by returning the updated *m*_*a*_. Merging two states *s*_*x*_ and *s*_*y*_ is done by simply changing both the source states of all outgoing transitions of *s*_*y*_ and the target states of all incoming transitions of *s*_*y*_ as *s*_*x*_. Note that a sliced model *m*_*sl*_ has only one final state as noted in Section 4.3.2, and therefore so does *m*_*a*_.

For example, let us consider the case where ***Append*** is called with parameters *m*_*a*_ = *slice*_1_ and *m*_*sl*_ = *slice*_2_ shown in the left block of Fig. 4. The algorithm merges *s*_9_ (i.e.,the final state of *m*_*a*_) and *s*_13_ (i.e., the initial state of *m*_*sl*_), resulting in $m_{a^{\prime }}$ shown in the right block of Fig. 4.

#### ***Union***

This algorithm takes as input a set of models *A*; it returns a model *m*_*u*_ that is able to accept all logs that can be accepted by all models in *A*. To do this, the algorithm simply merges the initial states of all models in *A*, and ends by returning the merged model as *m*_*u*_.

We remark that merging states in ***Append*** and ***Union*** can make the resulting model non-deterministic. Actually, the two algorithms are simplified^2^ versions of the standard NFA (Non-deterministic Finite Automata) concatenation and union operation, respectively. We will discuss non-determinism later in the determinization stage (see Section 4.4).

#### Application of ***Stitch*** to the running example

**Fig. 5:**
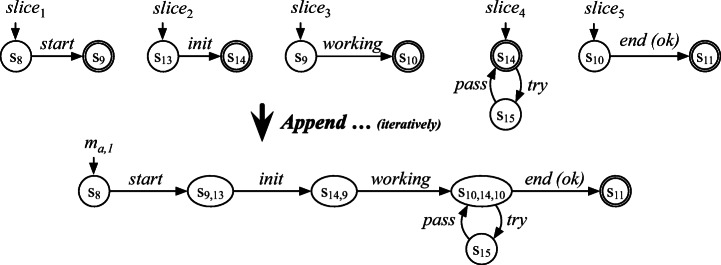
Illustration of building a system-level model for the running example log *l*_1_. The five sliced models are generated by ***Slice*** according to the partition of *l*_1_. They are appended by ***Append*** to build a system-level model *m*_*a*,1_ that accepts *l*_1_

**Fig. 6:**
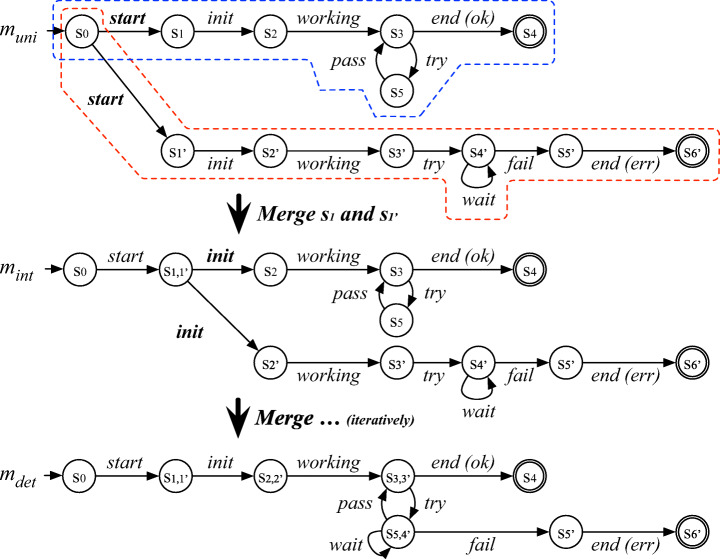
Illustration of the determinization using state merges for the system model build for the running example logs. The labels of nondeterministic transitions are highlighted in bold

Let us consider the case where the ***Stitch*** algorithm is called with parameters *L*_*sys*_ = {*l*_1_,*l*_2_} and *M*_*C*_ = {*m*_M_,*m*_J_}. For *l*_1_, the call to ***Partition*** yields $P = \langle l_{c,1}, l_{c,2}, \dots , l_{c,5} \rangle $ where *l*_*c*,1_ = 〈*e*_1,1_〉, *l*_*c*,2_ = 〈*e*_2,1_〉, *l*_*c*,3_ = 〈*e*_3,1_〉, *l*_*c*,4_ = 〈*e*_4,1_,*e*_5,1_,*e*_6,1_,*e*_7,1_〉, and *l*_*c*,5_ = 〈*e*_8,1_〉. For each *l*_*c*,*i*_ ∈ *P*, the call to ***Slice*** yields a sliced model *slice*_*i*_ shown in the top block of Fig. 5, originated from the component models *m*_M_ and *m*_J_ shown in Fig. 2. The five sliced models are appended to *m*_*a*_ using ***Append***, resulting in a system-level model *m*_*a*,1_ shown at the bottom of Fig. 5. The state names of *m*_*a*,1_ show how the initial and final states of the sliced models were merged. For example, *s*_10,14,10_ is generated by merging *s*_10_ (i.e., the final state of *slice*_3_), *s*_14_ (i.e., the initial and final state of *slice*_4_), and *s*_10_ (i.e., the initial state of *slice*_5_). Note that each *slice*_*i*_ accepts the corresponding *l*_*c*,*i*_ ∈ *P* and, as a result, *m*_*a*,1_ accepts *l*_1_. The algorithm ends the iteration for *l*_1_ by adding *m*_*a*,1_ into *A* and moves on to the next iteration to process log *l*_2_. After this second iteration completes, the newly built model *m*_*a*,2_ for *l*_2_ is added to *A*; the call to ***Union*** yields a system model *m*_*uni*_, shown at the top of Fig. 6. We can see that *m*_*uni*_ is composed of *m*_*a*,1_ (i.e., the upper subgraph enclosed with a blue dashed line) and *m*_*a*,2_ (i.e., the lower subgraph enclosed with a red dashed line).

In the above example, we can see that the output model *m*_*uni*_ accepts the input logs *L*_*sys*_ as expected. However, *m*_*uni*_ is actually not equivalent to *m*_S_ shown in Fig. 2a: there exist potential logs that only *m*_S_ can accept, but *m*_*uni*_ cannot. We will see how *m*_*uni*_ can be further transformed through the last stage of *PRINS*, i.e., determinization, described in detail in Section 4.4.

### Determinization

The last stage of *PRINS* post-processes the model yielded by the stitching stage for mainly converting a non-deterministic model into a deterministic one.

Through the projection, inference, and stitching stages, we already get a system model as an intermediate output. The non-determinism nature of such a model does not represent an issue in many use cases (e.g., program comprehension (Cook and Wolf 1998), test case generation Fraser and Walkinshaw, 2012). However, especially when a model is used as an “acceptor” of (the behavior recorded in) a log (e.g., in the case of anomaly detection Chandola et al., 2009), determinism is important for efficient checking. To broaden the use cases of *PRINS*, we propose this determinization stage as an optional post-processing in *PRINS*.

The simplest way of converting a non-deterministic model into a deterministic one is using standard algorithms, such as the powerset construction (Hopcroft et al. 2006), that guarantee the equivalence between the non-deterministic model provided in input and the deterministic one returned as output. However, the worst-case complexity of the powerset construction is exponential in the size of the non-deterministic model, making it an impracticable solution for many applications.

To tackle this issue, we introduce a new approach inspired by the heuristic-based determinization approach proposed by Damas et al. (2005). Unlike the powerset construction, their heuristic-based approach recursively merges the target states of non-deterministic transitions starting from the given state of the input model. While the idea of this approach is intuitive, since it simply merges states during the process of determinization, it may *generalize* the model being determinized, meaning the determinized model may accept additional logs that are not accepted by the original non-deterministic model. Our preliminary evaluation found that this simple strategy of merging states can produce an *over*-generalized model by merging too many states, especially when there are already many non-deterministic transitions in the input model. To avoid such over-generalization, we propose a new algorithm, called *Hybrid Determinization with parameter* *u* (HD_*u*_), by combining ideas from the heuristic-based determinization and the powerset construction methods.

Our HD_*u*_ merges the target states of non-deterministic transitions, similar to the heuristic-based determinization. However, to prevent over-generalization it applies a heuristic: HD_*u*_ does not merge a state with other states^3^ if the former has already been merged *u* times. The rationale behind this heuristic is to prevent the merging of too many states, which causes the over-generalization. If non-deterministic transitions remain because their target states are restricted from being merged because of the value of *u*, HD_*u*_ uses the powerset construction to remove the remaining non-determinism while preserving the level of the generalization. The larger the *u* value is used, the more the model can be generalized. The *u* value can also be seen as the weight between the heuristic-based determinization and the powerset construction; HD$_{\infty }$ is the same as the heuristic-based determinization, while HD_0_ is the same as the powerset construction.

Algorithm 3 shows the pseudocode of HD_*u*_. It takes as input a non-deterministic model *m*_*n*_ and a threshold *u*; it returns a deterministic model *m*_*d*_ that can accept all logs that can be accepted by *m*_*n*_. 
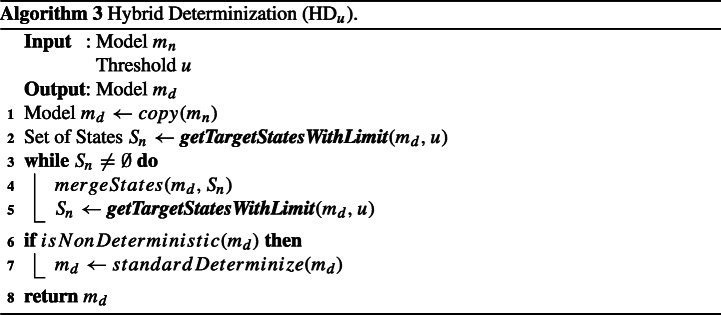


The algorithm iteratively merges the set of states *S*_*n*_ in *m*_*d*_ as determined by ***getTargetStatesWithLimit*** (described below) until it is empty (lines 3–5). After the iteration ends, if *m*_*d*_ is still non-deterministic, the algorithm removes all the remaining non-determinism using the powerset construction (lines 6–7). The algorithm ends by returning *m*_*d*_.

The heuristic to avoid the over-generalization is mainly implemented in function ***getTargetStatesWithLimit*** (whose pseudocode is shown in Algorithm 4). It takes as input a non-deterministic model *m*_*n*_ and a threshold *u*; it returns a set of states *S*_*n*_ to be merged to reduce non-determinism in *m*_*n*_, which does not contain the states that are restricted from being merged because of the threshold *u*. 
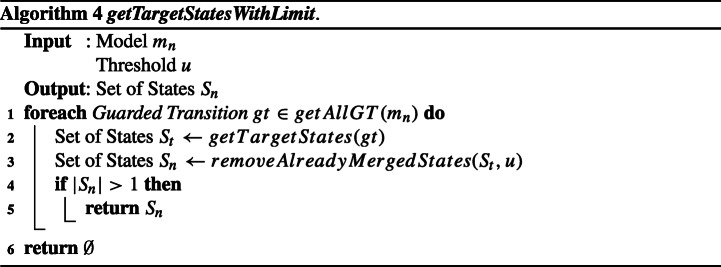


For each guarded transition *gt* in the transition relation of *m*_*n*_ (lines 1–5), the algorithm gets the set of target states *S*_*t*_ (line 2), removes the states that have already been merged *u* times from *S*_*t*_ to build *S*_*n*_ (line 3), and returns *S*_*n*_ (and ends) if it has more than one state (lines 4–5). If there is no such *S*_*n*_ for all guarded transitions, the algorithm ends by returning *S*_*n*_ = *∅* (line 6).

For example, let us consider the case where the ***getTargetStatesWithLimit*** algorithm begins the iteration for a non-deterministic (guarded) transition whose target states are *s*_*abc*_, *s*_*d*_, and *s*_*e*_, with *u* = 1. If *s*_*abc*_ was generated by merging three states *s*_*a*_, *s*_*b*_, and *s*_*c*_, *S*_*t*_ becomes {*s*_*abc*_,*s*_*d*_,*s*_*e*_} but *S*_*n*_ becomes {*s*_*d*_,*s*_*e*_} because *removeAlreadyMergedStates* excludes *s*_*abc*_ (since it has been already merged once, given *u* = 1). Since |*S*_*n*_| = 2, the algorithm ends by returning *S*_*n*_ = {*s*_*d*_,*s*_*e*_}.

Figure 6 shows how HD works for our running example. Recall that *m*_*uni*_ is the intermediate output of the stitching stage. Starting from the initial state, HD iteratively merges the target states of non-deterministic transitions, such as *s*_1_ and $s_{1}^{\prime }$ in *m*_*uni*_ and then *s*_2_ and $s_{2}^{\prime }$ in *m*_*int*_, until no more non-deterministic transition remains; the resulting model is *m*_*det*_. We can see that *m*_*det*_ is exactly the same as (i.e., is graph-isomorphic to) the ideal model *m*_S_ in Fig. 2.

Notice that HD_*u*_ causes a reduction in size of the models since it merges the target states of non-deterministic transitions in the course of its heuristic determinization. However, a more important question is to what extent the accuracy of the resulting models varies because of size reduction. In our empirical evaluation, we will assess the impact of using HD_*u*_ on the accuracy of the inferred models in *PRINS* with respect to the value of threshold *u*. We will also investigate the execution time of HD_*u*_ and devise practical guidelines for choosing the value of *u* (see Section 5).

## Evaluation

In this section, we report on the evaluation of the performance of *PRINS* in generating models of a component-based system from system logs.

First, we assess the execution time of *PRINS* in inferring models from large execution logs. This is the primary dimension we focus on since we propose *PRINS* as a viable alternative to state-of-the-art techniques for processing large logs. Second, we analyze how accurate the models generated by *PRINS* are. This is an important aspect because it is orthogonal to scalability but has direct implications on the feasibility of using the generated models to support software engineering tasks (e.g., test case generation). However, the execution time of *PRINS* and the accuracy of the models generated by *PRINS* might depend on its configuration, i.e., the number of parallel inference tasks in the inference stage (see Section 4.2) and the parameter *u* of HD_*u*_ in the determinization stage (see Section 4.4). Therefore, it is important to investigate the best configurations of *PRINS* before comparing it to state-of-the-art techniques.

Summing up, we investigate the following research questions:

- **RQ1:** *How does the execution time of* *PRINS* *change according to the parallel inference tasks in the inference stage?*
- **RQ2:** *How does the execution time of HD*_*u*_ *change according to parameter* *u*?
- **RQ3:** *How does the accuracy of the models (in the form of gFSMs) generated by HD*_*u*_ *change according to parameter* *u*?
- **RQ4:** *How fast is* *PRINS* *when compared to state-of-the-art model inference techniques?*
- **RQ5:** *How accurate are the models generated by* *PRINS* *when compared to those generated by state-of-the-art model inference techniques?*

### Benchmark and Settings

To evaluate *PRINS*, we assembled a benchmark composed of logs extracted from two sources: the LogHub project (He et al. 2020) and a personal computer (PC) running desktop business applications on a daily basis. Table 1 lists the systems we included in the benchmark (grouped by source) and provides statistics about the corresponding logs: the number of components (column *# Cmps*), the number of logs^4^ (column *# Logs*), the number of event templates (column *# Tpls*), and the total number of log entries^5^ (column *# Entries*).

**Table 1:** Subject systems and logs

Source	System	# Cmps	# Logs	# Tpls	# Entries	Conf
LogHub (He et al. 2020)	Hadoop	19	68	41	3575	1.00
	HDFS	8	1000	16	18741	0:95
	Linux	31	42	115	11259	0:78
	Spark	11	217	21	67725	0:98
	Zookeeper	18	36	40	25298	0:91
PC	CoreSync	54	1418	204	30223	0:94
	NGLClient	27	42	70	892	0.89
	Oobelib	12	250	147	56557	0:89
	PDApp	10	787	75	47394	0:87

LogHub (He et al. 2020) is a data repository containing a large collection of structured logs (and the corresponding event templates) from 16 different systems. Among them, we selected the logs of the five systems based on two conditions: (1) the component (name or ID) for each log entry is available in the logs; (2) the number of logs for each system is more than 10.

We set condition #1 because *PRINS* targets component-based systems; as for condition #2, we require a minimum number of logs to validate the accuracy as part of RQ2 (see Section 5.6).

To increase the diversity of our benchmark logs, we also included the logs of a personal computer running daily for office use. We collected the logs through the built-in Console.app application of macOS 10.15. Among the many logs available on the PC, we selected those fulfilling the same two conditions stated above, ending up with four systems. Additionally, to identify the events templates of the unstructured log messages in these logs, we first used state-of-the-art tools for log message format identification (i.e., Drain (He et al. 2017) and MoLFI (Messaoudi et al. 2018)) to compute an initial set of templates and then manually refined them, e.g., by collapsing similar templates into a single one. All the structured logs (anonymized to hide sensitive information) are available online (see Section 5.8).

To additionally evaluate whether the benchmark logs are sufficient to infer models that faithfully represent actual system behaviors, following another state-of-the-art model inference study (Emam and Miller 2018), we computed log confidence scores using the formula provided by Cohen and Maoz (2015). Briefly speaking, a low confidence score (e.g., ≤ 0.2) indicates that the logs are not sufficient, and therefore the model inferred from the logs are likely not to be compatible with the actual behaviors of the system under analysis. On contrary, a high confidence score (e.g., ≥ 0.85) indicates that the logs are probably sufficient for the inferred model to faithfully represent the actual system behaviors. Column *Conf* in Table 1 shows the confidence scores calculated for our benchmark logs. The values suggest that the logs are mostly sufficient to infer faithful models. Although the confidence score for Linux (0.78) is lower than the other benchmarks scores, the Linux logs are from an existing benchmark (He et al. 2020) and cannot therefore be improved. Furthermore, since our main focus is to compare *PRINS* and other model inference techniques using the same logs, having a somewhat moderate confidence score is not a major threat to the validity of our experiments.

We conducted our evaluation on a high-performance computing platform^6^, using nodes equipped with Dell C6320 units (2 Xeon E5-2680v4@2.4 GHz, 128 GB). We allocated four cores and 16 GB per job.

### RQ1: Parallel Inference

#### Methodology

To answer RQ1, we assessed the execution time of *PRINS* with different parallelization configurations for its inference stage. Specifically, we varied the maximum number of parallel workers (i.e., the maximum number of parallel inference tasks) from one to four in steps of one to investigate the relationship between the maximum number of parallel workers and the execution time of *PRINS*. For example, when the number is set to four, at most four workers are running in parallel to infer four component models at the same time in the inference stage of *PRINS*.

To infer individual component models in the inference stage of *PRINS*, we used MINT (Walkinshaw et al. 2016), a state-of-the-art model inference tool. We selected MINT because other tools are either not publicly available or require additional information other than just logs (e.g., source code or architectural design documents). In all experiments, we used the same configuration of MINT (i.e., minimum state merge score *k* = 2 and AdaBoost as data classifier algorithm), which we set based on the one used in a previous study (Walkinshaw et al. 2016) conducted by the authors of MINT.

For each system in our benchmark, we ran the four configurations of *PRINS* to infer a system model from the same logs and measured their execution time. To account for the randomness in measuring execution time, we repeated the experiment 10 times.

We remark that we disabled the determinization stage of *PRINS* because it is not the main focus of RQ1. Determinization configurations will be comprehensively investigated in RQ2 and RQ3.

#### Results

**Fig. 7:**
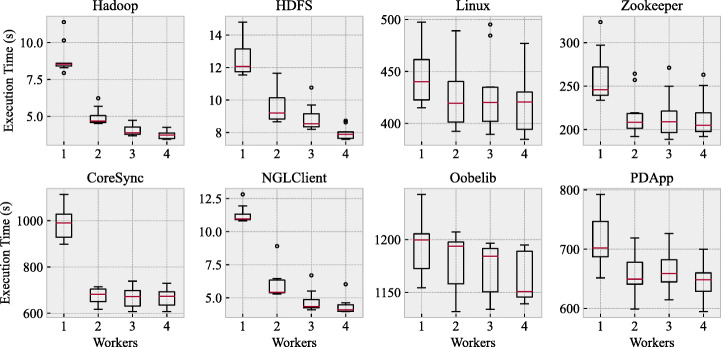
Relationship between the maximum number of parallel workers and the execution time of *PRINS*

Figure 7 shows the relationship between the maximum number of parallel inference tasks (workers) and the execution time of *PRINS*. None of the configurations was able to infer a model for Spark on all ten executions due to out-of-memory errors. This occurred because MINT (used by *PRINS* for inferring individual component models) could not process the (huge) log of a component that is responsible for producing about 97% of all log messages of the system.

For all systems in our benchmark, it is clear that execution time decreases as the maximum number of parallel inferences increases. This is consistent with the general expectation for parallelization.

However, doubling the maximum number of parallel inferences does not decrease the execution time in half. For example, there is no clear difference in execution time between two workers and four workers for Linux, Zookeeper, CoreSync, and PDApp. A detailed analysis of the results found that it is mainly because there are at most two major components that take up more than 70% of all log messages. For example, Linux has two major components that represent around 50% and 34% of all log messages, while the third-largest component takes up only 9.4% of all messages. This implies that, for systems like Linux, inferring component models is fast enough, except for a few major components, and therefore having more than three parallel workers does not significantly reduce execution time.

The answer to RQ1 is that the execution time of *PRINS* can be significantly reduced by the parallel inference of individual component models. However, the magnitude of the reduction in execution time is not linear with respect to the maximum number of parallel inferences, because not all components are equally sized in their logs. In practice, an engineer can set the maximum number of parallel inferences considering both available resources (e.g., the number of CPUs and the total size of memory) and the log size distribution of components.

### RQ2: Execution Time of Hybrid Determinization

#### Methodology

To answer RQ2, we assessed the execution time of HD_*u*_ with different parameter values for *u*. Specifically, we varied the value of *u* from one to ten in steps of one to investigate the relationship between the value of *u* and the execution time of HD_*u*_. To additionally compare HD_*u*_ to the standard powerset construction, we also set *u* = 0 (see Section 4.4 for more details).

For each system in our benchmark, we first ran *PRINS* without the determinization stage to infer a non-deterministic system model. *PRINS* internally used the same configuration of MINT as used in RQ1. For each non-deterministic model, we ran HD_*u*_ for all $u=0,1,\dots ,10$ and measured their execution time. To account for the randomness in measuring execution time, we repeated the experiment 10 times.

#### Results

**Fig. 8:**
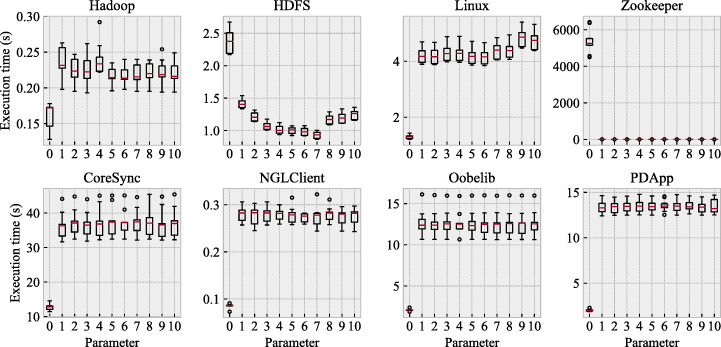
Relationship between the value of *u* and the execution time of HD_*u*_

Figure 8 shows the relationship between the value of *u* and the execution time of HD_*u*_. We have no results for Spark because its non-deterministic system model was not available for the reasons explained in Section 5.2.2.

For all the cases in which *PRINS* completed their execution for generating non-deterministic system models, HD_*u*_ (with *u* ≥ 1) took less than a minute. This implies that our hybrid determinization can efficiently determinize non-deterministic models generated by *PRINS*. On the other hand, the powerset construction (i.e., *u* = 0) took more than an hour for Zookeeper. This is due to the worst-case complexity of the powerset construction as discussed in Section 4.4. Interestingly, for the same non-deterministic model of Zookeeper, using *u* ≥ 1 significantly reduces the determinization time. This clearly highlights the benefit of hybrid determinization, i.e., combining the powerset construction and the heuristic-based determinization.

The answer to RQ2 is that the execution time of HD_*u*_ is practically the same for all *u* ≥ 1 because all of them are completed in less than a minute. This implies that the best value of *u* can be selected mainly based on the accuracy of the resulting models, which will be investigated in RQ3. On the other hand, the powerset construction is indeed very time-consuming in extreme cases, which is consistent with its well-known theoretical worst-case complexity. Since this cannot be predicted before running the determinization algorithms, in practice, we can conclude that HD_*u*_ with *u* ≥ 1 is to be recommended over the standard powerset construction.

### RQ3: Accuracy of Models Generated by Hybrid Determinization

#### Methodology

To answer RQ3, we first ran *PRINS* without the determinization stage to infer a non-deterministic system model for each system in our benchmark, as we did for RQ2. For each of the non-deterministic models, we then ran HD_*u*_ for all $u=0,1,\dots ,10$ and measured the accuracy of the deterministic models generated by HD_*u*_.

We measured the accuracy in terms of *recall*, *specificity*, and Balanced Accuracy (BA), following previous studies (Damas et al. 2005; Walkinshaw et al. 2016; Mariani et al. 2017; Emam and Miller 2018) in the area of model inference. Recall measures the ability of the inferred models of a system to accept “positive” logs, i.e., logs containing feasible behaviors that the system may exhibit. Specificity measures the ability of the inferred models to reject “negative” logs, i.e., logs containing behaviors that the system cannot exhibit. BA measures the balance between recall and specificity and provides the summary of the two.

However, it is intrinsically difficult to evaluate the accuracy of inferred models when there is no ground truth, i.e., reference models. To address this issue, we computed the metrics by using the well-known *k*-fold cross validation (CV) method with *k* = 10, which has also been used in previous model inference studies (Walkinshaw et al. 2016; Mariani et al. 2017; Emam and Miller 2018). This method randomly partitions a set of logs into *k* non-overlapping folds: *k* − 1 folds are used as “training set” from which the model inference tool infers a model, while the remaining fold is used as “test set” to check whether the model inferred by the tool accepts the logs in the fold. The procedure is repeated *k* times until all folds have been considered exactly once as the test set. For each fold, if the inferred model successfully accepts a positive log in the test set, the positive log is classified as True Positive (TP); otherwise, the positive log is classified as False Negative (FN). Similarly, if an inferred model successfully rejects a negative log in the test set, the negative log is classified as True Negative (TN); otherwise, the negative log is classified as False Positive (FP). Based on the classification results, we calculated ${recall}=\tfrac {|{TP}|}{|{TP}|+|{FN}|}$, ${specificity}=\tfrac {|{TN}|}{|{TN}|+|{FP}|}$, and the BA as the average of the recall and the specificity.

As done in previous work (Walkinshaw et al. 2016; Mariani et al. 2017; Emam and Miller 2018), we synthesized negative logs from positive logs by introducing small changes (mutations): (1) swapping two randomly selected log entries, (2) deleting a randomly selected log entry, and (3) adding a log entry randomly selected from other executions. The changes should be small, because the larger the change is, the easier an inferred model can detect the deviation of negative logs. To further increase the probability^7^ that a log resulting from a mutation contains invalid behaviors of the system, we checked whether the sequence of entries around the mutation location (i.e., the mutated entries and the entries immediately before and after the mutants) did not also appear in the positive logs.

#### Results

**Fig. 9:**
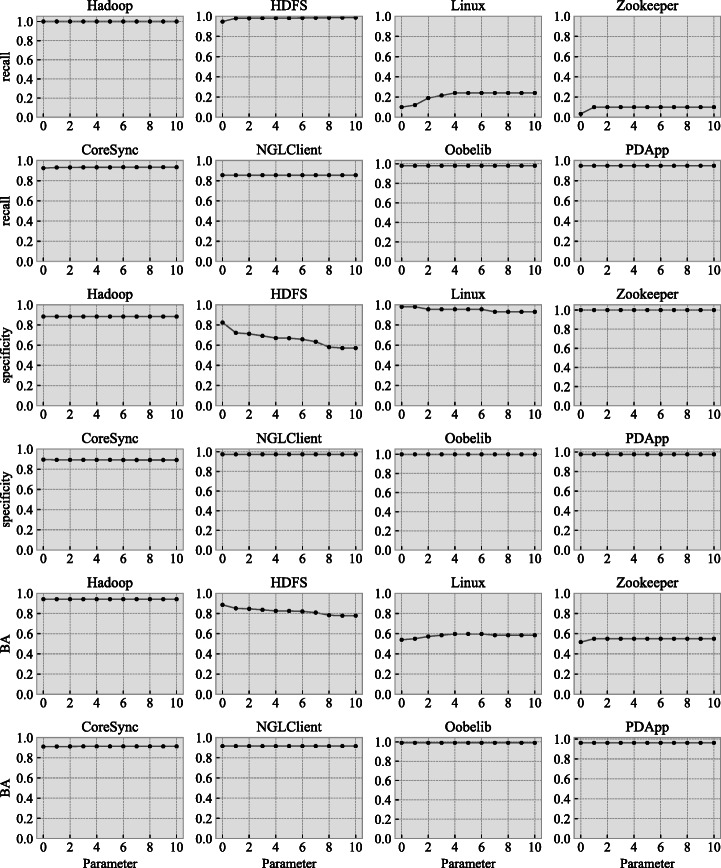
Relationship between the value of *u* and the accuracy of the models generated by HD_*u*_

Figure 9 shows the relationship between the value of *u* and the accuracy of the deterministic models generated by HD_*u*_. Again, Spark is not shown for the reasons explained in Section 5.2.2.

For Hadoop, CoreSync, NGLClient, Oobelib, and PDApp, there is no change in recall, specificity, and BA when the value of *u* changes. This means that, for these five systems, the accuracy of deterministic models generated by HD_*u*_ does not change when the value of parameter *u* changes (for $u=0,1,\dots ,10$). Furthermore, considering the fact that the powerset construction (i.e., HD_0_) guarantees the equivalence between the non-deterministic model provided in input and the deterministic one returned as output, identical accuracy for $u=0,1,\dots ,10$ also implies that, regardless of the value of *u*, HD_*u*_ can convert non-deterministic models into deterministic ones without sacrificing model accuracy for five out of the eight systems in our benchmark, regardless of the value of *u*.

For HDFS, Linux, and Zookeeper, as the value of *u* increases, recall values increase while specificity values decrease. This means that, for the deterministic models generated by HD_*u*_, if we increase the value of *u*, then the ability to correctly accept positive logs is improved whereas the ability to correctly reject negative logs is diminished. This is intuitive because increasing the value of *u* merges more states to remove non-deterministic transitions, yielding a generalized model that accepts more logs than the original, non-deterministic model.

However, we can distinguish the increase in recall and the decrease in specificity, because the former happens when the recall values of the input non-deterministic models are close to zero (i.e., Linux and Zookeeper), whereas the latter happens when the specificity values of the non-deterministic models are around 0.8. Since the non-deterministic models of Linux and Zookeeper were already incapable of correctly accepting positive logs, slightly improving them with determinization is not practically significant. In fact, the logs of Linux and Zookeeper were already inadequate for model inference in general, which will be discussed in detail in Section 5.6.2. On the other hand, the decrease in specificity for HDFS is significant for HD_*u*_ since it should preserve the high specificity of the non-deterministic model provided in input as much as possible. As a result, practically speaking, the smaller the value of *u*, the better. Indeed, this supports our idea that using *u* to limit over-generalization in hybrid determinization is helpful to avoid a significant accuracy loss.

The answer to RQ3 is that, for five out of eight systems in our benchmark, the value of *u* does not affect the accuracy of the deterministic models generated by HD_*u*_. However, for one system, the accuracy practically decreases as the value of *n* increases. Additionally considering the high execution time of HD_0_ (RQ2), we can therefore conclude that *u* = 1 is the best configuration trade-off for HD_*u*_ in terms of both execution time and accuracy in practice.

### RQ4: Execution Time of *PRINS* Compared to State-of-the-Art

#### Methodology

To answer RQ4, we assessed the scalability of *PRINS*, in terms of execution time, in comparison with MINT (Walkinshaw et al. 2016), the same tool that is used internally by *PRINS* to generate component-level models. In other words, we used *two* instances of MINT: the one used for the comparison in inferring system models; the other one used internally by *PRINS*. By doing this, we investigated to what extent the execution time of model inference can be improved by using the divide-and-conquer approach of *PRINS* compared to using a vanilla model inference.

Recall that *PRINS* can naturally infer many component models in parallel at the inference stage, which can further improve the execution time of *PRINS* as shown by the result of RQ1. To further investigate this aspect, we used *two* configurations of *PRINS*: *PRINS*-*P* where the parallel inference is enabled and *PRINS*-*N* where no parallelization is used. For *PRINS*-*P*, we set the maximum number of parallel inferences to four, based on the result of RQ1 and the number of allocated nodes (as described in Section 5.1). For both *PRINS*-*P* and *PRINS*-*N*, we used the determinization stage (i.e., HD_*u*_), since MINT produces a deterministic model. For the value of *u*, we used *u* = 1 based on the results of RQ2 and RQ3.

We also varied the size of input logs to better understand the impact of using larger logs on the execution time of *PRINS* and MINT. To systematically increase such size while preserving the system behaviors recorded in individual logs, we duplicated each of the logs following the experiment design of Busany and Maoz (2016). For example, when the duplication factor is set to eight for the 250 logs (56 557 log entries) of Oobelib, each of the 250 logs is duplicated eight times, and therefore a total of 250 × 8 = 2000 logs (8 × 56 557 = 452 456 log entries) are given as input both to *PRINS* and to MINT. Notice that the system characteristics, such as the number of components and the number of event templates, remain the same when using duplicated logs. Since MINT could not infer models for large logs due to out-of-memory failures or timeout (after 10 hours) in our preliminary evaluation, we only varied the duplication factor from 1 to 8 in steps of 1.

For each set of duplicated logs for each system in our benchmark, we ran MINT, *PRINS*-*P*, and *PRINS*-*N* to infer a deterministic system model from the same logs and measured the execution time of the tools. To account for the randomness in measuring execution time, we repeated the experiment three times and computed the average results.

#### Results

**Fig. 10:**
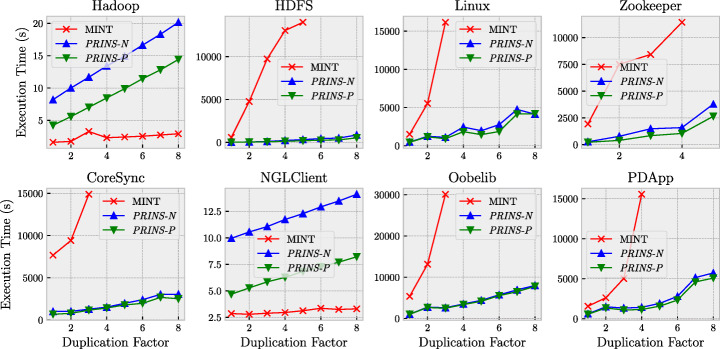
Comparison between MINT, *PRINS*-*P*, and *PRINS*-*N* in terms of execution time for various log sizes (obtained by varying the duplication factor of the benchmark logs)

Figure 10 shows the comparison results between MINT, *PRINS*-*P*, and *PRINS*-*N* in terms of execution time. Because MINT (both the standalone instance and the one used by *PRINS*) could not process the log of Spark, as explained in Section 5.2.2, we have no results for it. Also, due to the same reason, we have no results for Zookeeper with a duplication factor above 5.

For all the cases in which at least one of the tools completed their execution, we can see two distinct patterns in MINT’s execution time: for all of the duplicated logs, MINT completed its execution only in two cases (Hadoop and NGLClient) out of eight; otherwise, MINT could not complete its execution (HDFS, Linux, Zookeeper, CoreSync, Oobelib, and PDApp).

However, for Hadoop and NGLClient, for which MINT completed its execution for all of the duplicated logs, we can see that MINT was quite fast (the execution time is less than 5s); this can be attributed to the small size of the logs (28 600 entries for Hadoop and 7136 entries for NGLClient even with a duplication factor of 8). When the (standalone) MINT instance is already fast, *PRINS* is actually slower than MINT due to the overhead for projection, stitching, and determinization. Nevertheless, even in these cases, using *PRINS* instead of MINT is still practical since *PRINS*-*P* took less than 15s. Also, we want to remark that such small logs are not really representative of the large logs targeted by *PRINS*.

For the remaining six cases (HDFS, Linux, Zookeeper, CoreSync, Oobelib, and PDApp) with larger log sizes, the execution time of MINT increases steeply as the duplication factor increases. Furthermore, with a duplication factor above a certain value (5 for HDFS, 4 for Zookeeper and PDApp, and 3 for Linux, CoreSync, and Oobelib), MINT could not complete its execution due to out-of-memory failures (Zookeeper) or timeouts after 10 hours (HDFS, Linux, CoreSync, Oobelib, and PDApp). In contrast, for the same logs, the execution time of *PRINS*-*N* increases slowly as the duplication factor increases, and there is no case where *PRINS*-*N* could not complete its execution (except for Zookeeper in which the MINT instance internally used by *PRINS* for component model inference caused an out-of-memory failure when the duplication factor is greater than 5). This means that the scalability of model inference can be greatly improved by using the divide-and-conquer approach of *PRINS*. Furthermore, for Zookeeper with a duplication factor of 5, though MINT could not complete its execution due to out-of-memory failures, *PRINS*, on the other hand, completed successfully due to its divide-and-conquer approach.

Interestingly, for the six cases with larger log sizes, the difference between *PRINS*-*P* and *PRINS*-*N* is very small compared to the difference between *PRINS*-*N* and MINT. This means that the key factor in the scalability improvement of *PRINS* is the divide-and-conquer approach, not the parallel inference of component models.

The answer to RQ4 is that the divide-and-conquer approach of *PRINS* greatly improves the scalability of model inference for component-based system logs and can even enable model inference when MINT leads to out-of-memory failures.

### RQ5: Accuracy of *PRINS* Compared to State-of-the-Art

#### Methodology

To answer RQ2, we assessed the accuracy of the models inferred both by *PRINS* and by MINT for each system in our benchmark, using the same configuration for *PRINS* and MINT used as part of RQ4. We measured the accuracy in terms of recall, specificity and BA as we did for RQ3 (see Section 5.4.1 for more details).

#### Results

The accuracy scores of *PRINS* and MINT are shown in Table 2. Under the *Recall* column, sub-columns *M* and *P* indicate the recall of MINT and *PRINS*, respectively, and sub-column Δ_*R*_ indicates the difference in recall between *PRINS* and MINT in percentage points (pp). The sub-columns under the *Specificity* and *Balanced Accuracy* columns follow the same structure, with sub-column Δ_*S*_ indicating the difference in specificity between *PRINS* and MINT, and sub-column Δ_*B*_ indicating the difference in BA between *PRINS* and MINT. Again, none of the tools was able to infer a model for Spark, for the reasons explained in Section 5.5.2.

**Table 2:** Comparison between MINT (M) and *PRINS* (P) in terms of accuracy

		Recall			Specificity			Balanced accuracy		
System	M	P	ΔR	M	P	ΔS	M	P	ΔB LDS	
Hadoop	1.00	1.00	0.0	0.91	0.88	− 3.1	0.96	0.94	− 1.5	0.015
HDFS	0.98	0.98	− 0.1	0.37	0.72	34.9	0.68	0.85	17.4	0.007
Linux	0.36	0.12	− 23.5	0.89	0.98	9.5	0.62	0.55	− 7.0	0.561
Zookeeper	0.22	0.10	− 11.7	0.93	1.00	7.5	0.57	0.55	− 2.1	0.571
CoreSync	0.95	0.93	− 1.6	0.85	0.89	4.2	0.90	0.91	1.3	0.048
NGLClient	0.86	0.86	0.0	1.00	0.98	− 2.5	0.93	0.92	− 1.3	0.195
Oobelib	0.98	0.98	0.0	1.00	1.00	0.0	0.99	0.99	0.0	0.016
PDApp	0.97	0.95	− 2.3	0.98	0.98	− 0.1	0.97	0.96	− 1.2	0.014
Average	0.79	0.74	− 4.9	0.87	0.93	6.3	0.83	0.83	0.7	0.178

Differences between recall (Δ_*R*_), specificity (Δ_*S*_), and balanced accuracy (Δ_*B*_) values are expressed in percentage points (pp); ${\mathscr{L}}\mathcal {D}\mathcal {S}$ is the log-component diversity score

For all the cases in which the 10-fold CV completed without error, the average difference in BA between *PRINS* and MINT is only 0.7 pp, meaning that, on average, *PRINS* is as accurate as MINT in inferring system models in terms of BA. However, the average difference in recall between *PRINS* and MINT is -4.9 pp, while the average difference in specificity between *PRINS* and MINT is 6.3 pp. This implies that, on average, *PRINS* tends to infer models that are relatively less capable of accepting positive logs but more capable of rejecting negative logs than those inferred by MINT. The intuitive explanation is that a model built by *PRINS* could be, in certain cases discussed below, more specific to the flows of events recorded in individual input logs, due to the way *PRINS* builds the model. As described in Section 4.3, *PRINS* first builds an intermediate system-level model *for each execution log* and then merges these intermediate models by merging only their initial states at the end of the stitching. Though determinization after stitching might further merge the other states for removing non-determinism, it does so only for the states related to non-deterministic transitions. Therefore, the execution-specific flows of events captured in the intermediate system-level models can be maintained (without being merged with the others) in the final system model built by *PRINS*. In contrast, since MINT infers a model *for all system execution logs at once*, it tends to merge the execution-specific flows of events to a larger extent than *PRINS*. As expected, such characteristics also impact the size of inferred models. As shown in Table 3, the models inferred by *PRINS* have on average 3.6 times more states and 5.5 times more transitions than the models inferred by MINT. Since larger models are more difficult to manually analyze and comprehend, this might be interpreted as a drawback of *PRINS*. However, the models inferred by MINT are already too large to be manually analyzed and understood, especially for systems with large logs. Thus, automated techniques, such as model abstraction (Polyvyanyy et al. 2008), should be utilized in practice anyway. Furthermore, inferred models can be used for other important applications, such as test case generation (Fraser and Walkinshaw 2012) and anomaly detection (Chandola et al. 2009), which do not require minimally sized models. Therefore, the increased model size can be considered acceptable given the significant scalability improvement reported in Section 5.5.

**Table 3:** Comparison between MINT and *PRINS* in terms of model size

	States			Transitions		
System	MINT	*PRINS*	ratio	MINT	PRINS	ratio
Hadoop	67	65	1.0	70	66	0.9
HDFS	76	392	5.2	177	1308	7.4
Linux	342	1990	5.8	476	3322	7.0
Zookeeper	376	3184	8.5	553	10667	19.3
CoreSync	3876	7524	1.9	4318	10798	2.5
NGLClient	148	154	1.0	160	195	1.2
Oobelib	447	1195	2.7	545	1484	2.7
PDApp	1301	3523	2.7	1466	3801	2.6
Average	829	2253	3.6	971	3955	5.5

Looking at the results for individual systems, results differ significantly in terms of Δ_*R*_ and Δ_*S*_ and it is important to understand why to draw conclusions. For instance, for HDFS, the value of Δ_*S*_ is high (34.9pp), while the value of Δ_*R*_ is negligible. This shows that *PRINS*, compared to MINT, can significantly increase the accuracy of the inferred models by increasing their ability to correctly reject negative logs, without compromising their ability to correctly accept positive logs.

On the other hand, for Linux and Zookeeper, the values of Δ_*R*_ are negative and practically significant (-23.5pp for Linux and -11.7pp for Zookeeper) while the values of Δ_*S*_ are positive and practically significant as well (34.9pp for Linux and 7.5pp for Zookeeper). Furthermore, the recall values of both MINT and *PRINS* are relatively lower for Linux and Zookeeper compared to the recall values for the other systems. In terms of the 10-fold CV, this means that the positive logs in the test set are not properly accepted by the models inferred from the logs in the training set for Linux and Zookeeper. Experimentally, this is mainly due to the logs in the training set being *too different* from the logs in the test set, this being caused by the highly diverse logs of Linux and Zookeeper overall. From a practical standpoint, this implies that, regardless of the model inference technique, a model inferred from existing logs may not be able to correctly accept unseen (but positive) logs if the latter are too different from the former. However, for the reasons mentioned above, the issue of highly diverse logs has a moderately larger impact on *PRINS* than on MINT. Practical implications are discussed below.

Before running model inference, to effectively predict and avoid cases where *PRINS* is likely to be worse than MINT and where both techniques fare poorly, we propose a new and practical metric to measure the diversity of logs. Our log diversity metric is based on the combination of components appearing in the individual logs because (1) *PRINS* targets component-based systems considering not only the individual components’ behaviors but also their interactions, (2) it is much simpler than using, for example, the flows of log entries in the logs, and (3) it does not require any extra information other than the logs. More formally, let *L* be a set of logs of a system and let *C*(*l*) be the set of components appearing in a log *l* ∈ *L*. We define *log-component diversity score* (${\mathscr{L}}\mathcal {D}\mathcal {S}$) of the system logs *L*_*sys*_ as ${\mathscr{L}}\mathcal {D}\mathcal {S}(L_{{sys}}) = \frac {U-1}{N-1}$, where $U = \lvert \{ C(l) \mid l\in L_{{sys}} \} \rvert $ (i.e., the total number of unique *C*(*l*)s for all *l* ∈ *L*_*sys*_) and $N = \lvert L_{{sys}}\rvert $ (i.e., the total number of logs in *L*_*sys*_). In other words, ${\mathscr{L}}\mathcal {D}\mathcal {S}$ indicates the ratio of logs that are unique (i.e., different from the others) in terms of the set of components appearing in the individual logs, ranging between 0 and 1; the higher its value, the higher the diversity of the logs in terms of recording different component interactions. For instance, ${\mathscr{L}}\mathcal {D}\mathcal {S}(L_{\texttt {S}})=0$ for our running example logs *L*_S_ = {*l*_1_,*l*_2_} because *N* = 2 and $U= \lvert \{C(l_{1}), C(l_{2})\}\rvert = 1$ (since *C*(*l*_1_) = *C*(*l*_2_) = {Master,Job}). This means that *L*_S_ is not diverse at all in terms of the appearing components. Notice that ${\mathscr{L}}\mathcal {D}\mathcal {S}$ is a characteristic of logs, which can be calculated before model inference takes place.

We measured ${\mathscr{L}}\mathcal {D}\mathcal {S}$ for the logs of each system in our benchmark. Column ${\mathscr{L}}\mathcal {D}\mathcal {S}$ in Table 2 shows the results. We can see that the resulting ${\mathscr{L}}\mathcal {D}\mathcal {S}$ values of Linux (0.561) and Zookeeper (0.571) are much higher than those of the other systems, which range between 0.007 (HDFS) and 0.195 (NGLClient). This confirms that ${\mathscr{L}}\mathcal {D}\mathcal {S}$ can be effectively used to predict whether the models inferred from the existing logs can correctly accept unseen (but positive) logs or not before running model inference.

In practice, if ${\mathscr{L}}\mathcal {D}\mathcal {S}$ is high (e.g., > 0.2) for the logs of a system, it implies that these logs do not sufficiently exercise, in a comprehensive way, the potential behaviors of the system. As a result, there is a high probability that many component interactions have not been recorded or too rarely so. Therefore, an engineer can address this problem by collecting more system logs until ${\mathscr{L}}\mathcal {D}\mathcal {S}$ is low enough.

The answer to RQ5 is that, compared to MINT, *PRINS* tends to infer models that are more capable of rejecting negative logs (i.e., yielding a higher specificity value) while sometimes being less capable of accepting positive logs (i.e., yielding a lower recall value). The latter happen anyway only in cases where logs are not adequate for both techniques to work well. In practice, an engineer can compute the diversity score of the logs before running model inference, and easily determine whether more logs should be collected, either through testing or usage, until the score is acceptable.

### Discussion and Threats to Validity

From the results above, we conclude that *PRINS* is an order of magnitude faster than MINT in model inference for component-based systems, especially when the input system logs are large and the individual component-level logs are considerably smaller than the system logs, without significantly compromising the accuracy of the models. Furthermore, since the large majority of modern software systems is composed of many “components”, which can be modules, classes, or services, depending on the context, the logs typically encountered in practice will satisfy the best conditions for *PRINS* to fare optimally: the system logs are large but the individual component-level logs are considerably smaller. There are situations where *PRINS* exhibits a poorer recall than MINT. However, this is the case when the system logs are inadequate for model inference in general, regardless of the technique, and we have proposed a way to detect such situations and remedy the problem.

One drawback of the divide-and-conquer approach in *PRINS* is the increased size of inferred models. In this sense, *PRINS* can be seen as sacrificing model size for improving the execution time of model inference. Nevertheless, it is worth to note that *PRINS* does not significantly compromise the accuracy of the inferred models. Furthermore, given the significant execution time reduction in model inference on large logs, increasing model size can be considered acceptable.

In terms of threats to validity, using a specific model inference tool (MINT) is a potential factor that may affect our results. However, we expect that applying other model inference techniques would not change the trends in results since the fundamental principles underlying the different model inference techniques are very similar. Furthermore, MINT is considered state-of-the-art among available tools. Nevertheless, an experimental comparison across alternative tools would be useful and is left for future work.

We used *k*-fold cross validation to evaluate the accuracy of inferred models due to the lack of ground truth (i.e., reference models) for our benchmark systems. Therefore, the computed accuracy scores might not faithfully represent the similarity between the inferred models and their (unknown) ground truths, especially when the collected logs do not sufficiently represent the system behaviors. To mitigate this issue, we calculated the log-confidence values, following existing studies, and these results suggested that the logs in our benchmarks are sufficient to derive faithfully inferred models. Furthermore, since the same logs are used for both *PRINS* and MINT, not relying on ground truth does not severely affect our empirical evaluation results.

### Data Availability

The implementation of *PRINS* is available as a Python program. The replication package, including the benchmark logs and our implementation of *PRINS*, is at https://github.com/SNTSVV/PRINS.

## Related Work

Starting from the seminal work of Biermann and Feldman (1972) on the *k-Tail* algorithm, which is based on the concept of state merging, several approaches have been proposed to infer a Finite State Machine (FSM) from execution traces or logs. *Synoptic* (Beschastnikh et al. 2011) uses temporal invariants, mined from execution traces, to steer the FSM inference process to find models that satisfy such invariants; the space of the possible models is then explored using a combination of model refinement and coarsening. *InvariMINT* (Beschastnikh et al. 2015) is an approach enabling the declarative specification of model inference algorithms in terms of the types of properties that will be enforced in the inferred model; the empirical results show that the declarative approach outperforms procedural implementations of *k-Tail* and *Synoptic*. Nevertheless, this approach requires prior knowledge of the properties that should hold on the inferred model; such a pre-condition cannot be satisfied in contexts (like the one in which this work is set) where the knowledge about the system is limited and the only information about the system is provided by logs. *mk-Tails* (Busany et al. 2019) is a generalization of the *k-Tail* algorithm from single to many parameters, which enables fine-grained control over the abstraction (generalization) on different subsets of the events. It allows users to deal with the trade-off between size and accuracy in model inference.

Other approaches infer other types of behavioral models that are richer than an FSM. *GK-tail+* (Mariani et al. 2017) infers guarded FSM (gFSM) by extending the *k-Tail* algorithm and combining it with Daikon (Ernst et al. 2007) to synthesize constraints on parameter values; such constraints are represented as guards of the transitions of the inferred model. *MINT* (Walkinshaw et al. 2016) also infers a gFSM by combining EDSM (Evidence-Driven State Merging) (Cheng and Krishnakumar 1993) and data classifier inference (Witten et al. 2016). EDSM, based on the Blue-Fringe algorithm (Lang et al. 1998), is a popular and accurate model inference technique, which won the Abbadingo (Lang et al. 1998) competition; it is also utilized in DFASAT (Heule and Verwer 2013) that won the StaMinA competition (Walkinshaw et al. 2013). Data-classifier inference identifies patterns or rules between data values of an event and its subsequent events. Using data classifiers, the data rules and their subsequent events are explicitly tied together. *ReHMM* (Reinforcement learning-based Hidden Markov Modeling) (Emam and Miller 2018) infers a gFSM extended with transition probabilities, by using a hybrid technique that combines stochastic modeling and reinforcement learning. ReHMM is built on top of MINT; differently from the latter, it uses a specific data classifier (Hidden Markov model) to deal with transition probabilities.

Model inference has also been proposed in the context of distributed and concurrent systems. *CSight* (Beschastnikh et al. 2014) infers a communicating FSM from logs of vector-timestamped concurrent executions, by mining temporal properties and refining the inferred model in a way similar to *Synoptic*. *MSGMiner* (Kumar et al. 2011) is a framework for mining graph-based models (called Message Sequence Graphs) of distributed systems; the nodes of this graph correspond to Message Sequence Chart, whereas the edges are determined using automata learning techniques. This work has been further extended (Kumar et al. 2012) to infer (symbolic) class level specifications. However, these approaches require the availability of channel definitions, i.e., which events are used to send and receive messages among components.

Liu and Dongen (Liu et al. 2016) use a *divide-and-conquer* strategy, similar to the one in our *PRINS* approach, to infer a system-level, hierarchical process model (in the form of a Petri net with nested transitions) from the logs of interleaved components, by leveraging the calling relation between the methods of different components. This approach assumes the knowledge of the caller and callee of each component methods; in our case, we do not have this information and rely on the *leads-to* relation among log entries, computed from high-level architectural descriptions and information about the communication events.

Nevertheless, all the aforementioned approaches cannot avoid scalability issues due to the intrinsic computational complexity of inferring FSM-like models; the minimal consistent FSM inference from logs is NP-complete (Gold 1967) and all the more practical approaches are approximation algorithms with polynomial complexity.

One way to tackle the intrinsic scalability issue of (automata-based) model inference is to rely on distributed computing models, such as MapReduce (Dean and Ghemawat 2008), by transforming the sequential model inference algorithms into their corresponding distributed version. In the case of the *k-Tail* algorithm, the main idea (Wang et al. 2015) is to parallelize the algorithm by dividing the traces (sequences of log messages) into several groups, and then run an instance of the sequential algorithm on each of them. A more fine-grained version (Luo et al. 2017) parallelizes both the trace slicing and the model synthesis steps. Being based on MapReduce, both approaches require to encode the data to be exchanged between mappers and reducers in the form of key-value pairs. This encoding, especially in the trace slicing step, is application-specific; for instance, to correctly slice traces recorded by an online shopping system, different event parameter values, such as user id, order id, and item id, must be correctly identified and categorized from individual messages beforehand. Notice that this is more challenging than just identifying parameter values from free-formed messages, since different types of parameters must be distinguished. Hence, MapReduce cannot be used in contexts in which the system is treated as a black-box, with limited information about the data recorded in the log entries. Furthermore, though the approach can infer a FSM from large logs of over 100 million events, the distributed model synthesis can be significantly slower for *k* ≥ 3 (of *k-Tail*), since the underlying algorithm is exponential in *k*.

Another way of taming scalability is to reduce the size of input logs by sampling them from the entire set of collected logs using statistical analysis and provide statistical guarantees on the inferred models. This is called *statistical log analysis* and was first presented by Busany and Maoz (2016). Its key idea is to iteratively sample new logs until the probability of adding new system behaviors into the model inferred by sampled logs is less than a given level of confidence threshold. While the idea of using statistical analysis to address the scalability of model inference is promising, as already noted by the authors, it is only applicable to *sequential* model inference algorithms, where each log can be processed independently (Busany and Maoz 2016). *PRINS*, on the other hand, is applicable to all model inference algorithms as only the inference target is changed from systems to components. Therefore, all model inference algorithms can benefit from using the divide-and-conquer approach in *PRINS*.

## Conclusion

In this paper, we addressed the scalability problem of inferring the model of a component-based system from system logs, assuming that the only information available about the system is represented by the logs. Our approach, called *PRINS*, first infers a model of each system component from the corresponding logs; then, it merges the individual component models together taking into account the flow of events across components, as reflected in the logs. Our evaluation, performed on logs from nine datasets, has shown that *PRINS* can process large logs an order of magnitude faster than a publicly available and well-known state-of-the-art technique without significantly compromising the accuracy of inferred models. While there are some cases where *PRINS* achieves a moderately lower recall than the state-of-the-art, this happens when the logs are inadequate for model inference in general, regardless of the technique. Furthermore, we have proposed an easy way to detect such cases and remedy the problem.

As part of future work, we plan to evaluate *PRINS* on different datasets, especially collected from real-world industrial applications, and to integrate it with other model inference techniques. We also aim to assess the effectiveness of the inferred models when applied to support software engineering activities, such as test case generation.

## Footnotes

1 A deterministic gFSM *m* = (*S*,*E**T*,*G*,*δ*,*s*_0_,*F*) with *δ* : *S* × *E**T* × *G* → *S* can be easily converted into a deterministic FSM $m'=(S, {\Sigma }, \delta ^{\prime }, s_{0}, F)$ with $\delta ^{\prime }: S\times {\Sigma } \to S$ where Σ = *E**T* × *G*.

2 To be precise, merging two states is not equivalent to introducing an epsilon-transition from one to another, but equivalent to introducing bi-directional epsilon-transitions between the two states.

3 Regardless of the number of states to be merged, multiple states can be merged into one state at once, not incrementally.

4 In LogHub (He et al. 2020), the original dataset for HDFS contains 575061 logs (distinguishable by block-ids), but we used only 1000 logs that are randomly sampled from all logs, since we found that the 1000 logs are representative enough, as they contain all event templates that appear in all logs.

5 When additional logging level information (e.g., info, warn, debug, error) was available for each log entry, we only used the log entries of the two main levels, i.e., info and error, as the others provide unnecessary details (e.g., the status of a specific internal variable) for building behavioral models.

6 The experiments presented in this paper were carried out using the HPC facilities of the University of Luxembourg (Varrette et al. 2014) (see https://hpc.uni.lu for more details).

7 Recall that there are no reference models for the subject systems, and therefore we cannot verify if a synthesized log correctly contains an invalid system behavior.
